# Effect of Tannins Addition on Thermal Stability of Furfurylated Wood

**DOI:** 10.3390/polym15092044

**Published:** 2023-04-25

**Authors:** Mahdi Mubarok, Elham Azadeh, Firmin Obounou Akong, Stéphane Dumarçay, Philippe Gérardin, Christine Gérardin-Charbonnier

**Affiliations:** 1 LERMAB (Laboratoire d’Etudes et de Recherche sur le Matériau Bois), Université de Lorraine, INRAE, 54000 Nancy, France; mahdi.mubarok@yahoo.fr (M.M.);; 2 Department of Forest Products, Faculty of Forestry and Environment, IPB University, Bogor 16680, Indonesia

**Keywords:** furfuryl alcohol, leaching resistance, reticulation, tannin, thermal stability, wood modification

## Abstract

This article presents the effect of the addition of condensed tannins, used as a reticulation agent, on the polymerization of furfuryl alcohol during wood furfurylation, as well as the effect of these condensed tannins on the thermal stability of modified wood. Three kinds of dicarboxylic acids (adipic acid, succinic acid, and tartaric acid), as well as glyoxal, used as model of a wood reticulation agent, were used to catalyze the polymerization of furfuryl alcohol or tannin-furfuryl alcohol solutions. Impregnation of furfuryl alcohol or tannin-furfuryl alcohol solution into the wood, followed by curing at 103 °C for a specific duration, was performed for the wood modification. The thermal stability of the obtained tannin-furfuryl alcohol polymers and their corresponding modified woods was investigated. The leaching resistance and dimensional stability of the modified woods were also evaluated. Results indicated that the partial substitution of furfuryl alcohol by the tannins improved the polymerization reactivity in conditions where furfuryl alcohol alone did not lead to the formation of a solid polymeric material. The thermal stability and leaching resistance of the furfurylated wood in the presence of tannins were improved. Dimensional stability was also improved for furfurylated samples, but the effect of tannin addition was not so obvious, depending on the acidic catalyst used.

## 1. Introduction

Tannins, a well-known polyphenolic compound extracted from different tree species, have use in several applications, including adhesives, composites, pharmaceuticals, medicines, and foods and beverage products. Due to their chemical structure, tannins are often considered as an alternative to phenol, produced using petrochemistry, for the preparation of thermoset resins. However, due to their low reactivity with wood, tannins, and especially condensed tannins, necessitate the addition of cross-linkers or other reactive chemicals or additives to develop wood modification treatments [1,2,3,4]. Utilization of a high weight ratio of tannins towards the cross-linkers and low concentration of the impregnating solution to modify wood lead to only small improvement in the properties of modified wood, particularly in terms of dimensional stability, leaching resistance, and decay durability. Tannin/furanic thermoset resins have been reported as biosourced plastics or abrasive grinding wheels for different applications [5,6,7]. Although the reaction between tannins and furfuryl alcohol (FA) has been studied for more than three decades [8], its use for the modification of solid wood is still very limited, thus triggering further investigations to develop more environmentally friendly non biocide treatments for wood modification.

Furfurylation is an important wood modification technology already in use on an industrial scale [9,10,11,12]. Related to this type of treatment, and considering that the polymerization of furfuryl alcohol depends on several factors, including the nature and the concentration of the catalyst used and the concentration of FA solution [13,14,15], it seems interesting to evaluate the effects of the addition of condensed tannins as a reticulating agent, and of different types of acids as catalysts, to improve the properties of modified wood. The effect of such treatments on the mechanical properties and decay durability of modified woods have been already described in a previous study [16]. The aim of the present paper is to report the effects of the additions of tannins on the polymerization of furfuryl alcohol and on the thermal stability of the polymer. This effect was also investigated on some properties of the impregnated wood samples, including their dimensional thermal stability.

## 2. Materials and Methods

### 2.1. Impregnating Solutions and Resulting Polymers

The impregnating solution was produced according to the formulation described in Table 1. Distilled water, adipic acid (AA), succinic acid (SA), tartaric acid (TA), furfuryl alcohol (FA), glyoxal (Glyox), and condensed tannins (procyanidin) powder extract (Tan) (Sylvateam, Italy) were formulated in a weight percent concentration. The pH value of each impregnating solution was recorded.

A Curing test at 103 °C was performed for each impregnating solution through the evaporation and curing of each impregnating solution using a polypropylene plastic cup with a similar solution quantity in an oven for 24 h. The resulting furfuryl alcohol or tannin-furfuryl alcohol polymer was then retrieved from the plastic cup for further characterization.

### 2.2. Wood Modification

European beech (*Fagus sylvatica*), an abundant hardwood species in Europe with a low dimensional stability and poor durability against the wood degrading organisms, was used in this study. The wood samples (dried density, ±687 kg.m^−3^), measuring 20 mm (L) × 20 mm (R) × 10 or 5 mm (T), were carefully dried to avoid crack formation at increasing temperatures (80 °C, 24 h; 103 °C, 24–48 h), and weighed (m_0_); the volumes were then measured (v_0_). The samples, aside from the untreated wood (A), were thereafter inserted into different impregnating solutions under vacuum conditions (100 KPa, 1 h), followed by pressure conditions (1200 KPa, 30 min). At the end of the impregnation process, all samples were removed and the excess solution on the samples was wiped off with a paper towel. Each sample was then measured to determine its wet weight (m_1_). The samples were then dried at room temperature for 3 days before curing at 103 °C for 48 h. Each cured sample was re-measured for its weight (m_2_) and volume (v_2_).

Solution uptake after impregnation was calculated according to Equation (1):

Solution uptake (%) = 100 × (m_1_ − m_0_)/m_0_ (1)

where m_0_ is the initial mass of the oven-dry wood at 103 °C and m_1_ is the wet mass of wood just after impregnation.

Weight percent gain (WPG) and bulking (B) were calculated after curing according to Equations (2) and (3):

WPG (%) = 100 × (m_2_ − m_0_)/m_0_ (2)

B (%) = 100 × (v_2_ − v_0_)/v_0_ (3)

where WPG is the weight percent gain, B is the bulking value, and m_2_ and v_2_ are the dry mass and dry volume of impregnated wood sample after curing process, respectively.

Fixation of the resin into the wood structure was evaluated after a leaching test, carried out according to NF X 41-568 [17]. Treated and untreated wood samples (5 mm × 20 mm × 20 mm, n = 18–30) were placed in different flasks containing a volume of water at least five-fold their volume. Flasks were placed on a plate stirrer and subjected to different leaching periods (1, 2, 4, 8, 16, and 48 h) with a change of water between each period. The wood samples were kept without water for 16 h after the period of 4 h. At the end of the leaching periods, the wood samples were dried at 103 °C for 48 h and weighed (m_3_). The quantity of resin leached out from the wood was calculated according to Equation (4).

L (%) = 100 × (m_2_ − m_3_)/m_2_ (4)

where L is the leaching value of the wood sample and m_3_ is the dry mass of the wood after leaching process.

Dimensional stability, measured as anti-swelling efficiency (ASE), was measured using a three-cycle drying-wetting system. Wood samples (10 mm (L) × 20 mm (R) × 20 mm (T), 7 replicates) were dried at 103 °C for 48 h before determination of their dry volume (V_d_). Samples were then immersed in distilled water in different 100 mL beakers according to whether they had been initially treated or not. The beakers were placed in a desiccator and subjected to vacuum conditions (10 kPa) for 1 h before restoration of atmospheric pressure and kept submerged in the water at room conditions for 23 h. This drying-wetting cycle was repeated for the following second and third cycles. The volume of each wet wood sample was then measured again (V_w_). Swelling and ASE were calculated according to Equations (5) and (6).

S (%) = 100 × (V_w_ − V_d_)/V_d_ (5)

where S is the percentage of swelling of wood sample, V_d_ the dry volume of the sample, and V_w_ the wet volume of the sample.

ASE (%) = 100 × (S_0_ − S_1_)/S_0_ (6)

where ASE is the percentage of anti-swelling efficiency of treated wood, S_0_ the swelling of untreated wood at cycle 1, and S_1_ the swelling of treated wood after the third cycle.

### 2.3. Thermogravimetric Analysis (TGA)

A thermal property of the polymer and the wood samples was performed by means of a TGA/DSC1-TMA/SDTA 84Xe instrument from Mettler Toledo, equipped with STARe v.11 fr System program. Ten milligrams (±1.00 mg) of each sample powder were placed on an alumina crucible. The thermal property test of each sample was launched according to the following step:

1. T = 25 to 103 °C, t = 8 min, air condition (v = 0 mL/min);
2. T = constant at 103 °C, t = 7.8 min, air condition (v = 0 mL/min);
3. T = 103 to 700 °C (for wood samples) or 900 °C (for polymer), t = 59.7 or 79.7 min, N_2_ condition (v = 50 mL/min);
4. T = 700 °C or 900 °C to 25 °C, t = 67.5/87.5 min, N_2_ condition (v = 50 mL/min).

## 3. Results and Discussion

### 3.1. Polymerizationof Resin Alone

**Figure 1:**
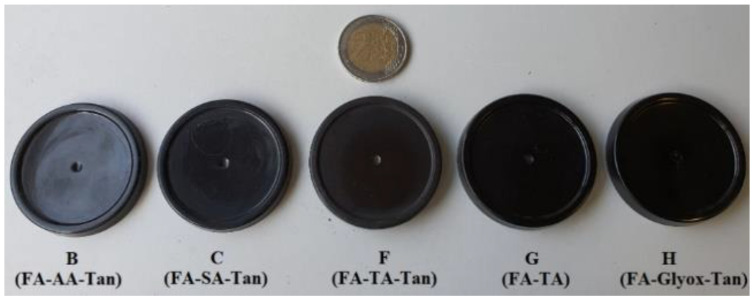
Furfuryl alcohol/Furfuryl alcohol-tannin based polymers.

The curing tests of the impregnating solutions at 103 °C for 24 h during preliminary testing demonstrated that almost all formulated solutions polymerized completely, creating a black colored solid polymer (Figure 1). However, due to the lower reactivity of adipic acid (AA) and succinic acid (SA) when compared to tartaric acid (TA), the system that contained only FA-AA (D) or FA-SA (E) did not lead to a solid polymeric material at the given curing temperature. These results were in agreement with previous research works on the characteristics of some catalysts during FA polymerization; these studies reported that SA (K_1_ 6.5 × 10^−5^) or AA (K_1_ 3.72 × 10^−5^) have lower dissociation constants than TA (K_1_ 9.6 × 10^−4^) [13], hence providing lower reactivity towards polymerization of FA. On the other hand, a partial substitution of FA by the tannins into solutions D and E in order to become solution B and C improved reactivity of the polymerization reaction, indicating there was a synergistic effect due to this tannin addition, since a solution of FA without the addition of tannins did not polymerize without the addition of acid (SA or AA, in this case) or vice versa.

### 3.2. Polymerizationof Resin within the Wood Structure

During wood impregnation, solution uptake of different impregnating solutions presented approximately similar values (>100%) (Table 2). After the curing process, due to higher reactivity of TA (higher dissociation constant) than SA in these furanic or tannin-furfuryl alcohol polymerizations, WPG values of the treatments using TA (F and G) were higher than the treatments using SA. Further, it seemed that the addition of tannins could increase both the WPG values and the bulking values for some treatments. There was an exception for treatment F; this was due to the higher reactivity of TA than other acids. As such, the addition of the tannin increased the reactivity of the impregnating solution, thereby intensifying the curing/cross-linking process. Therefore, due to this intensive cross-linking process in treatment F, the final wood volumes after the curing process were slightly smaller than the corresponding treatment without tannin addition (G), thereby decreasing their bulking values. The effect of the addition of tannins on dimensional stability is less obvious. The addition of tannins allowed the slight improvement of the dimensional stability of modified wood for formulations catalyzed with adipic and succinic acid, while the dimensional stability was lower for formulation catalyzed with tartaric acid. Replacement of the acidic catalyst with glyoxal allowed to good dimensional stability to be obtained, which remained lower than that of furfuryl alcohol formulations catalyzed by tartaric acid.

### 3.3. Thermogravimetric Analysis (TGA)

**Figure 2:**
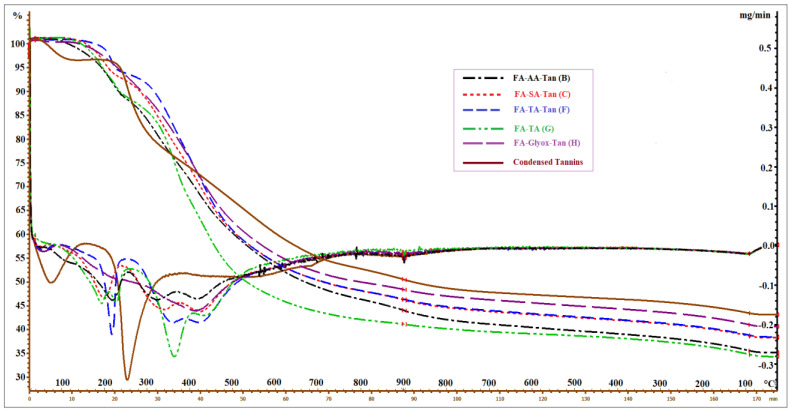
TG (**top**) and DTG (**bottom**) of different furanic or tannin-furfuryl alcohol polymers and condensed tannins.

The TGA profile shows differences in the thermal stability property of all solid polymers (B, C, F, G, and H) (Figure 2, Table 3). Among these polymers, polymer G (FA-TA) showed the highest mass loss value, indicating that thermal stability of a solely furanic-based polymer (polymer G) was lower than most of the tannin-furfuryl alcohol polymers (especially polymer C, F, and H). The effect of tannin addition and its extensive reticulation with FA might also trigger these differences, improving the thermal stability of the matrix tannin-furfuryl alcohol polymer. Tannins contain aromatic structures that have higher thermal stability than any other created polymers presented in this study. However, due to the lower reactivity of AA towards the reticulation of the tannin-furfuryl alcohol matrix, polymer B presented the highest mass loss values among the tannin-furfuryl alcohol polymers.

**Figure 3:**
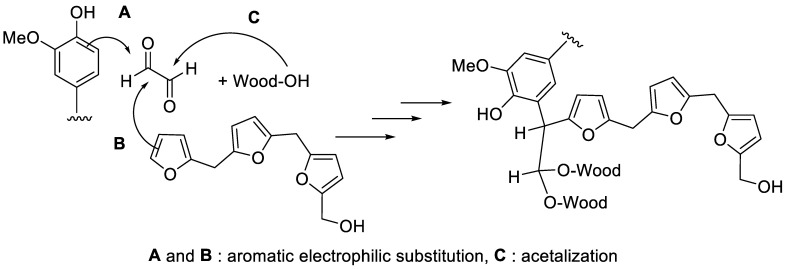
Possible crosslinking mechanisms of glyoxal.

Differential thermal gravimetry (DTG) studies confirmed different thermal properties of the polymers (Figure 2, Table 3). These studies show that dicarboxylic acid-catalyzed tannin-furfuryl alcohol polymers (B, C, F) underwent three stages of thermal degradation. Due to the different characteristics of the acids used, stage I and II provided higher differences among these polymers, especially in terms of their inflection temperature (T_inf_) and mass loss rate. However, due to different cross-linking mechanisms resulting from the addition of glyoxal (Figure 3), involving either aromatic electrophilic substitutions with aromatic moieties of lignin of polyfurfuryl alcohol or acetalization with hydroxyl groups of wood, polymer H presented obviously different characteristics than the other samples due to the reticulation reactions between glyoxal with the tannins [3] when compared to other systems based on acid-catalyzed tannin-furfuryl alcohol polymers. Thermal degradation of polymer H took place primarily during a single stage, at a lower mass loss rate and a higher degradation temperature, producing higher char residue during measurement. On the other hand, the absence of tannins in polymer G produced a different profile of its thermal property, disclosing four stages of thermal degradation. A close T_inf_ appearing between stage I (~192 °C) and II (~228 °C) occurred due to a possible first and second step of chain scission, involving chain scission of some weaker bonds (e.g., ester bonds) formed during the reaction of tartaric acid-catalyzed polymerization of furfuryl alcohol. Further, a pronounced degradation of polymer G occurred at ~360 °C [18]. A partial substitution of furfuryl alcohol by the tannins (a single T_inf_ of tannins ~253 °C) in formula G generated a polymer with a slightly higher thermal stability (polymer F), increasing the initial temperature of thermal decomposition of the polymer and increasing the amount of char produced. Above 400 °C, total degradation of all polymers occurred, yielding final residues of thermally stable char.

**Figure 4:**
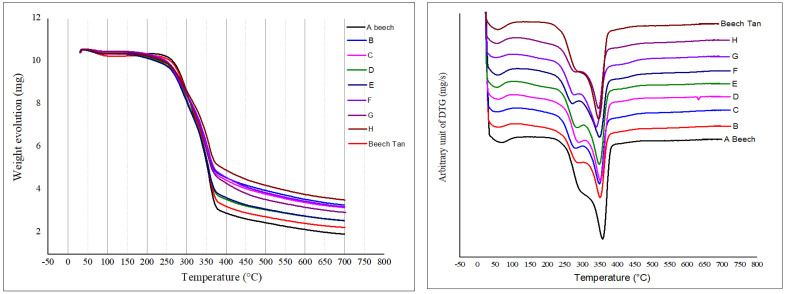
TGA (**left**) and DTG (**right**) curves of untreated wood vs treated wood.

With an almost similar thermogravimetric property to the untreated wood, the wood modified with either furanic or tannin-furfuryl alcohol polymer underwent earlier thermal degradation at ~122 to 275 °C, possibly due to evaporation of water as a byproduct of the incomplete polycondensation of the polymer, the chain scission of some weaker bonds, and degradation of some wood components (Figure 4).

Further degradation occurred between 275 and ~310 °C (depending on the impregnated polymer), corresponding mostly to the degradation of some wood components and part of the impregnated polymer (Figure 2). Similar to the untreated wood, the highest mass loss rate occurred between ~375 and 492 °C, whereby both the wood components and the impregnated polymers decreased considerably due to thermal degradation of the remaining polymer and carbonization. Since the thermal stability of the impregnated furanic or tannin-furfuryl alcohol polymer was higher than that of the untreated wood, all modified woods, according to their obtained average WPG, demonstrated greater thermal stability than the untreated woods (Table 4). As previously explained, the addition of tannins, which have high thermal stability due to their aromatic structures, contributes to the higher thermal stability of the tannin-furfuryl alcohol modified wood when compared to the untreated wood. For the impregnated furanic polymer, the effect of the wood component modification (e.g., modification of lignin) was the main reason for the thermal stability improvement of the modified wood when compared to the untreated wood.

**Figure 5:**
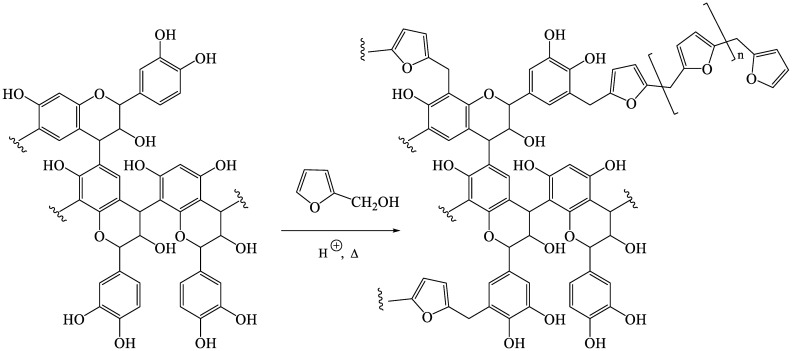
Potential reactions of furfuryl alcohol with condensed tannins.

Based on the results, a thermal stability order of all wood samples could be arranged as follows: untreated Beech < D-treated wood < E-treated wood < Beech-Tan < G-treated wood < B-treated wood < C-treated wood < F-treated wood < H-treated wood (Table 4, Figure 4). Although the WPG values of the modified wood in treatments D (21.3 ± 2.5%) and E (27.0 ± 1.5%) were higher than that in Beech-Tan (9.3 ± 0.6%, *n* = 7), it showed that the thermal stability of the tannin-impregnated wood was slightly higher than the wood impregnated with polymers D or E. However, at a higher WPG level, the wood modified with treatment G (WPG = 38.4 ± 1.1%) presented higher thermal stability than tannin-impregnated beech (Beech-Tan). Therefore, the WPG and the type of the impregnated polymer were the main factors influencing the thermal stability of the modified wood. In addition, even though Beech-Tan presented a higher thermal stability than untreated wood and showed an additional increase in the thermal stability of tannin-furfuryl alcohol modified wood (especially treatments B and C when compared to treatments D and E, as well as Beech compared to Beech-Tan (Table 4)), the disappearance of a specific single tannin peak in the DTG of the tannin-furfuryl alcohol based polymer (Figure 2) indicated that the tannins reacted completely with the other components in their constructed polymers, thereby creating the new thermal stability property of the polymer. Potential reactions between furfuryl alcohol polymers and aromatic moiety of tannins or lignin are proposed in Figure 5, explaining the disappearance of tannins signals in DTG and higher leaching resistance of treatments performed in the presence of tannins.

Other reactions involving esterification reactions of hydroxyl groups of wood components or tannins with different carboxylic acids used as catalysts could also be involved in the polymerization reaction at high temperatures.

## 4. Conclusions

The partial substitution of FA by the tannins, either during polymerization of FA or wood furfurylation in the acidic medium, provided various advantages. It allowed the polymerization of reactants into a hard-solid polymer regardless of the catalyst used, while polymerization of furfuryl alcohol alone was not always possible. The thermal stability, dimensional stability, and leaching resistance of the furfurylated wood were improved. Through this tannin addition, polymerization of the tannin-furfuryl alcohol solution could occur even under an acidic medium with a lower dissociation constant, generating tannin-furfuryl alcohol polymer and improving the properties of the modified wood. FA modified wood in the presence of tannins presented higher thermal stability when compared to furfurylated wood, which presented a higher thermal stability than the beech control. However, due to a black polymer characteristic obtained from this tannin-furfuryl alcohol system, the modified wood has a darker color than its corresponding furfurylated wood. Nevertheless, this renewable modification technology might stimulate the application of the constructed polymers or the modified woods for other advanced research work or other special products. Due to the improvements of properties as a result of the addition of tannins, tannin-furfuryl alcohol modified wood can be used for classical applications of furfurylated wood, including for decking, flooring, siding, or cladding for exterior uses.

## Figures and Tables

**Table 1:** Formulation of the applied impregnating solution and their pH values.

ID	Impregnating Solution	pH
A	Untreated (without impregnation)	6.8 (water)
B	AA 2.2% + FA 44.4% + Tan 8.9% + water 44.4%	3.2
C	SA 2.2% + FA 44.4% + Tan 8.9% + water 44.4%	2.8
D	AA 2.2% + FA 53.3% + water 44.4%	4.0
E	SA 2.2% + FA 53.3% + water 44.4%	3.6
F	TA 2.2% + FA 44.4% + Tan 8.9% + water 44.4%	2.0
G	TA 2.2% + FA 53.3% + water 44.4%	2.0
H	Glyoxal 20% + FA 40% + Tan 8% + water 32%	2.9

**Table 2:** Wood characteristic during and after wood modification process.

ID	Solution Uptake (%) *	WPG (%) *	Bulking (%) *	Leaching (%) **	ASE Cycle III (%) ***
A	-	-	-	1.5 ± 0.2	9.2 ± 5.9
B	120.1 ± 5.0	36.2 ± 2.4	13.2 ± 2.1	3.8 ± 0.6	46.1 ± 2.5
C	121.4 ± 7.0	39.4 ± 0.7	15.0 ± 1.6	2.2 ± 0.3	49.8 ± 2.2
D	120.1 ± 5.4	21.3 ± 2.5	8.1 ± 1.4	5.4 ± 1.3	39.5 ± 3.7
E	116.1 ± 6.4	27.1 ± 1.5	11.8 ± 1.0	5.2 ± 0.9	47.5 ± 3.0
F	118.5 ± 6.3	46.2 ± 2.4	13.8 ± 0.8	2.0 ± 0.1	44.6 ± 1.8
G	124.2 ± 2.8	38.4 ± 1.1	17.8 ± 0.9	2.8 ± 0.2	66.1 ± 7.9
H	121.4 ± 7.2	50.8 ± 4.4	14.9 ± 1.0	2.1 ± 0.3	51.1 ± 3.0

* Each value was based on the average of 10 samples; ** Each value was based on the average of 18–30 samples (leaching method referred to NF X 41-568 [17]; *** Each value was based on the average of 7 samples (ASE test used drying-wetting method).

**Table 3:** TGA and DTG data of furanic or tannin-furfuryl alcohol polymers and condensed tannins.

Treatment	Sample	TGA	The Main T_inf_ in DTG (°C)			
		Total ML (%)	Stage I	Stage II	Stage III	Stage IV
B	FA-AA-Tan	64.65	221	324	420	-
C	FA-SA-Tan	61.53	200	338	425	-
F	FA-TA-Tan	61.26	216	360	423	-
G	FA-TA	64.98	192	228	364	435
H	FA-Glyox-Tan	58.74	418	-	-	-
Additional	Tannins	56.54	253	-	-	-

Total ML is the total mass loss during analysis TGA; T_inf_ the inflection temperature.

**Table 4:** Mass loss data of untreated wood and treated wood recorded from the TGA.

Treatment	Sample	% MLTGA
A	Beech	88.75
B	FA-AA-Tan-Beech	79.04
C	FA-SA-Tan Beech	77.13
D	FA-AA Beech	85.30
E	FA-SA Beech	83.69
F	FA-TA-Tan-Beech	76.63
G	FA-TA Beech	79.64
H	FA-Glyox-Tan-Beech	74.44
Additional	Beech-Tan	82.51

## Data Availability

Not applicable.
